# 

**Published:** 1863-11

**Authors:** 


GENTLEMEN OF THE DENTAL PROFESSION:—

Enclosed you have a meagre account of the first Dental Convention ever assembled in this State. It must be apparent to every Dentist that associations of this kind are productive of much good. They are the means of forming friendly relations with each other; of imparting knowledge, gained by individual experience, to the whole number. Here mind comes in contact with mind, and dissimilar views are discussed and decided upon. Different modes of practice are debated, and judgment rendered for the best. Fees are compared and a standard of prices adopted. An ESPRIT DU CORPS raised, creating an honorable ambition to excel. Considering then, the importance of sustaining and making useful the "IOWA STATE DENTAL SOCIETY," we appeal to each and every Practitioner in the State to come forward at our next meeting, and show his interest in the welfare of our Profession by his presence. To those who *can not* be present, and yet wish to join our Association, we say, send a dollar to the Secretary with the request that your name be signed to the Constitution and By-Laws.

We hope that many of you will furnish written articles on subjects pertaining to the Profession.

The next regular meeting of this Society will be holden at Iowa City, on Wednesday at 9 o'clock, A. M, January 20th, 1864.

A. J. McGARVEY,
SECRETARY.

---

### Editorial.

### WESTERN NEW YORK DENTAL SOCIETY.

WE had the pleasure of meeting with the Western New York Dental Association at its regular annual meeting held at Buffalo on the second Tuesday of October last.

There were present over fifty dentists of Western New York. The meeting continued two days, and was full of interest. All the members appeared to be interested in the success of the movement, and desirous that good should result from the deliberations; and from the manner in which the meeting was conducted it could not be otherwise. Two essays were read, both of which we have published.

The discussions partook of a practical character, and elicited the views and modes of practice of those present very fully. We cheerfully acknowledge the receipt of many new and good ideas, for which we thank the whole Association.

There was a movement put into operation which we deem rather important, and one which should be adopted by similar societies everywhere. It consisted of the appointment of a committee whose duty it shall be, so far as possible, to procure the history of Dentistry from its beginning, in Western New York, and also biographical sketches; more especially professional, of course—of the pioneers. The Committee was requested to procure a suitable book into which all this should be inscribed, which book shall be the property of the society and kept in its archives.

Though our profession is of so recent origin, the beginning will soon be beyond our reach, if some means are not employed to save it from almost total obscurity. At this meeting the social element was well cultivated, which is certainly a very material part of our Associations. Though this Society is large, it will still grow. T.

### PITTSBURGH DENTAL SOCIETY.

We were blessed with the opportunity of making a visit to this Association at its November meeting, held in the city of Pittsburgh. It was an exceedingly gratifying visit.

The profession of Pittsburgh and its vicinity is certainly wide awake, and working with all the zeal of vigorous youth.

They have established a Dental Institute, which is well sustained. It is the first and only one in the United States, independent of the dental Colleges. There are several objects in view in its establishment. Here the poor, or those not able to avail themselves of the services of a good dentist in a private office, can secure reliable treatment and operations. It can be used as a counteracting and controling influence, to some extent at least, upon those who would vampyre-like, prey upon the profession that gave them birth, suck its life-blood, tear out its vitals, and drag it down to the dust as a vile thing, if they can but fatten upon its destruction. (We propose to express ourselves on this matter ere long.)

This society holds its meetings in the Institute. It is neatly fitted up, as much so as most offices. There consultations may be made, and experiments conducted.

At this meeting a very excellent essay was read by Dr. J. D. White, on "Anæsthetics," in connection with which much interesting discussion took place.

A Committee was appointed for the immediate procurement of an apparatus for the manufacture and administration of nitrous oxide gas, as an anæsthetic, the experiments to be conducted in the Institute. The profession in Pittsburgh will make a landmark. T.

### AUTOMATIC MALLET.

We have had a slight experience with this instrument, and think better of it than before; some things can be very well accomplished by it. The force and precision of the stroke can be perfectly regulated by the will of the operator.

The operator will very soon become familiar with the instrument. The one we refer to is made by Mr. Helman, of Brown's Dental Depot notoriety. He has them for sale. T.

### NEW DENTAL DEPOT IN CINCINNATI.

### JAMES LESLIE,

The long established Manufacturer of

### LESLIE'S CELEBRATED GOLD FOIL.

Desires to return his thanks to the Dental Profession for the liberal support afforded to him since extending his business; to include every article of Dental supplies. Among other arrangements for the season and year just opened, I desire particularly to call the attention of my Dental friends to the following

### REGULINE GOLD FOIL,

A new article and highly approved.

Spaldings Dental Record and Account, Spaldings Appointment Book; published by A. M & J. Leslie. Agent for Dr. Geo. E. Hayes Vulcanizing Oven, American Hard Rubber Co. Gum and Gutta Percha; Dental Furniture; Forceps, &c., &c.

No. 126 Fourth street, North West corner of Fourth and Race streets, opposite the Office of the Cincinnati Commercial. White's Teeth, Alloys. and Watt's Foil, always on hand.

